# Predicted 25-hydroxyvitamin D over the adult lifetime and the risk of ovarian cancer

**DOI:** 10.1093/aje/kwae070

**Published:** 2024-05-17

**Authors:** Jennifer A Ritonja, Coraline Danieli, Magnoudewa Priscille Pana, Michael J Palmer, Kevin L’Espérance, Vikki Ho, Michal Abrahamowicz, Anita Koushik

**Keywords:** ovarian cancer, case-control studies, 25-hydroxyvitamin D, vitamin D, weighted cumulative exposure model

## Abstract

The evidence from previous studies of serum 25-hydroxyvitamin D (25(OH)D) and ovarian cancer risk is not conclusive. However, the 25(OH)D levels were generally only measured in late adulthood, which may not capture the etiologically relevant exposure periods. We investigated predicted 25(OH)D over the adult lifetime in relation to ovarian cancer risk in a population-based case-control study conducted from 2011 to 2016 in Montreal, Canada (*n* = 490 cases and 896 controls). Predicted 25(OH)D was computed using previously validated regression models. Unconditional multivariable logistic regression models were used to estimate adjusted odds ratios (aORs) and 95% CIs for average predicted 25(OH)D over the adult lifetime and ovarian cancer risk. In addition, the relative importance of different periods of past 25(OH)D exposure was explored using a weighted cumulative exposure (WCE) model. For each 20-nmol/L increase in average predicted 25(OH)D over the adult lifetime, the aOR (95% CI) was 0.73 (0.55-0.96). In WCE analyses, the inverse association was strongest for exposures 5 to 20 years and 35 to 55 years prior to diagnosis, with aORs (95% CIs) of 0.82 (0.69-0.94) and 0.79 (0.66-1.02), respectively, for each 20-nmol/L increase in predicted 25(OH)D. These results support an inverse association between 25(OH)D levels in adulthood and ovarian cancer risk.

**This article is part of a Special Collection on Gynecological Cancers**.

Ovarian cancer is the fifth-leading cause of cancer death among Canadian and US women, and is by far the deadliest of all gynecologic cancers.^1^ Unfortunately, in most cases, the disease has spread beyond the pelvis at diagnosis,^1^ reducing the long-term success of treatment,^2^ and currently available screening modalities do not reduce mortality.^3^ Prevention is thus of critical importance, which necessitates the identification of modifiable risk factors.

Higher vitamin D exposure, measured directly in serum or indirectly through its main sources of sunlight and diet, has been associated with reduced incidence and mortality for multiple cancers.^4^ Evidence from observational studies is strongest for colorectal cancer; associations with cancers of the breast, prostate, and ovary have been less consistent.^4^ Results from the Vitamin D and Omega-3 Trial (VITAL), a randomized placebo-controlled trial, showed that daily high-dose vitamin D supplementation (>2000 IU/d) did not reduce overall invasive cancer incidence.^5^ However, a secondary analysis of VITAL found that vitamin D supplementation reduced the incidence of advanced (metastatic or fatal) cancers.^6^

Experimental research in ovarian cancer models shows that vitamin D metabolites inhibit ovarian cancer cell growth,^7^^,^^8^ increase apoptosis and the expression of the vitamin D receptor,^9^ and delay the development and progression of chemically induced ovarian tumors in vivo and in vitro.^10^ However, the findings from epidemiologic research are mixed and not conclusive.^11^^,^^12^ A feature of most previous studies is that the vitamin D measure did not encompass all sources. For instance, 8 of 17 studies systematically reviewed recently relied on dietary vitamin D exposure only and thus ignored differences in sun exposure.^11^ Interestingly, a reduced ovarian cancer risk was suggested in 4 of the 5 studies that examined current or past sun exposure as a proxy for vitamin D.^11^

Studies of serum 25-hydroxyvitamin D (25(OH)D),^13^^‑^^22^ a biomarker that measures total vitamin D exposure from all sources, have mostly reported a lack of association,^17^^‑^^22^ although some found that higher 25(OH)D was associated with a reduced ovarian cancer risk.^13^^‑^^16^ Although serum 25(OH)D reflects total vitamin D exposure from both dietary (food and supplements) and sun exposure, most existing studies may still be limited because baseline blood collection was in late adulthood (~50-58 years) and thus did not capture earlier exposures.^13^^‑^^22^ Indeed, early adulthood exposures of parity and oral contraceptives are established ovarian cancer risk factors,^23^ thus, it is possible that the induction period for a potential protective effect of vitamin D occurs over decades. Interestingly, recent Mendelian randomization studies suggest that a genetically higher 25(OH)D level is associated with a reduced ovarian cancer risk, supporting a role of vitamin D.^20^^,^^24^^,^^25^

Overall, there is strong biological plausibility for a relation between vitamin D and ovarian cancer, but further epidemiologic studies are needed that capture vitamin D from all sources across the adult lifetime to detect the etiologically pertinent periods. The best available biomarker for total vitamin D exposure is serum concentration of 25(OH)D^26^; however, its measurement is expensive and essentially impossible to obtain retrospectively. A cost-efficient alternative is to use models that predict serum 25(OH)D based on self-reported lifestyle and personal attributes for different life periods.^27^^‑^^29^ Using previously validated prediction models of serum 25(OH)D,^29^ we examined predicted 25(OH)D levels over the adult lifetime in relation to epithelial ovarian cancer risk in a population-based case-control study.

## Methods

### Study overview

The Prevention of Ovarian Cancer in Quebec (PROVAQ) study is a population-based case-control study of female Canadian citizens aged 18-79 years conducted in Montreal, Canada, from 2011 to 2016.^30^ Among 652 incident cases of histologically confirmed epithelial cancer of the ovary, fallopian tube, or peritoneum from 7 Montreal hospitals, 507 patients (78%) agreed to participate. Nine cases were later identified as nonepithelial or metastatic, for a total of 498 cases. Tumors were classified according to behavior (borderline or invasive), histology (serous, mucinous, endometroid, or clear cell), and grade (I-IV). Population controls were identified from the Quebec electoral list and recruited in an approximate 2:1 ratio to cases, frequency-matched to cases by 5-year age group and Montreal region.^30^ Among 1634 eligible women invited to serve as controls, 908 (56%) agreed to participate.

All cases and controls participated in an in-person interview during which information on personal and family medical history, and sociodemographic, reproductive, and lifestyle characteristics, including multiple vitamin D determinants, was collected for each year during the entire adulthood. A calendar charting major life events was used to aid recall.^31^ Cases were interviewed, on average, 4.8 months after diagnosis (5th-95th percentiles, 2.2-8.9 months). Ethical approval was provided by the Research Ethics Committee of the Centre de recherche du CHUM and the participating hospitals, and all participants provided written informed consent.

### Exposure

In a previous PROVAQ study, multivariable linear regression models that predict 25(OH)D levels were developed and validated among a sub-sample of 200 control participants, who provided blood samples for measurement of serum 25(OH)D concentrations.^29^ The final model for vitamin D supplement users included alcohol intake, supplement dose, menopausal status, outdoor time, recent sun vacation, and sun protection as predictors, whereas for supplement nonusers, predictors were body mass index (BMI; weight (kg)/height (m)^2^), sun sensitivity, season of blood sample collection, recent sun vacation, and sun protection.^29^ In cross-validation analyses, the models explained 46%-47% of the variance in gold standard measurements of serum 25(OH)D levels in an independent sample drawn from the same population.^29^

In the present study, we applied these validated models to all PROVAQ participants according to their questionnaire responses to 25(OH)D predictors, obtained for each year of life throughout adulthood, starting at age 20 years. Missing data were minimal for 25(OH)D predictors (0%-4%), thus simple imputation methods were used. Appendix S1 provides details on the prediction models, their application, and missing data imputation.

Average predicted 25(OH)D level over the adult lifetime was calculated as the simple mean of the yearly predicted 25(OH)D from age 20 years to 2 years prior to the index date, defined as the diagnosis date for cases and the interview date for controls. The last 2 years were not included in the calculation, to account for latency between disease initiation and diagnosis and to minimize the risk of potential reverse-causality bias. In separate analyses, average predicted 25(OH)D was modeled as either a continuous variable or dichotomized as adequate (≥50 nmol/L) versus inadequate (<50 nmol/L), based on the US Institute of Medicine dietary reference intakes for vitamin D.^32^ No participants were predicted to be within the deficient (<30 nmol/L) and concerningly high (≥125 nmol/L) ranges.^32^

### Statistical analysis

Unconditional multivariable logistic regression was used to estimate adjusted odds ratios (aORs) and 95% CIs for the association between average predicted 25(OH)D over the adult lifetime and ovarian cancer risk. A directed acyclic graph identified the minimally sufficient confounder set of age (continuous), educational attainment (less than high school, high school, college or technical school, university), ancestry (French Canadian, other European, or other/mixed ancestry), parity (nulliparous, 1-2, ≥3 births), moderate-to-vigorous physical activity across all domains (ie, transport, occupation, housework, and recreation) averaged over the adult lifetime (continuous, in metabolic equivalent hours per week [MET-h/wk]), and BMI at age 20 years (<25, ≥25) (Figure S1). For continuous measures of predicted 25(OH)D and continuous covariates (i.e., age and total moderate-to-vigorous physical activity), the linearity of their associations with the logit of the probability of ovarian cancer was assessed using multivariable fractional polynomials, based on likelihood ratio tests at a 2-tailed α = .05.^33^ For continuous predicted 25(OH)D, aORs were estimated for each increment of 20 nmol/L, corresponding to the expected 25(OH)D increase among healthy adults with a daily intake of 1000-1250 IU of vitamin D_3_ supplements.^34^^,^^35^

We examined whether associations between predicted 25(OH)D and ovarian cancer overall varied by (1) menopausal status, (2) BMI 2 years prior to study participation, (3) average BMI over the adult lifetime, and (4) ever versus never use of vitamin D supplements, by including product terms between the potential effect modifier and predicted 25(OH)D in the logistic regression models. Interaction on the additive scale was assessed by calculating the relative excess risk due to interaction and their corresponding 95% CIs.^36^^‑^^38^

Multivariable polytomous logistic regression was used to estimate aORs (95% CIs) for borderline versus invasive ovarian cancers, and for individual histological types (serous, mucinous, endometroid, and clear cell). Given that ovarian cancer etiology is thought to differ by both tumor behavior and histological type,^39^ analyses by histological type were conducted for invasive and borderline cancers in 2 separate models. We also estimated aORs for the high-grade serous carcinoma histotype, the most common ovarian cancer type, using unconditional logistic regression.

To account for possible measurement error due to the use of an estimated, rather than actual, measure of 25(OH)D concentration, we used the simulation-extrapolation (SIMEX) approach,^40^ using the prediction errors estimated while validating our original models.^29^ With this method, increasing increments of measurement error in predicted 25(OH)D were simulated, and new resulting ORs were calculated. We then fit a linear model between the added variance and the estimated ORs, and then extrapolated this relation back to where there was no measurement error (variance = 0), the intercept of which gives the OR corrected for potential measurement error. To account for the potential impact of nonparticipation, we fit logistic regression models for the binary outcome of participation (yes/no), among case and control participants separately, using age, education level, parity, smoking status and duration, and engagement in sun-seeking behaviors, collected from both nonparticipants and participants, as predictors. We then weighted the main logistic regression analyses by the inverse of the individual predicted probabilities of participation (more detail is provided in Appendix S2).^41^^,^^42^

To explore the relative importance of predicted 25(OH)D exposure during different periods over the adult lifetime course, we used the flexible weighted cumulative exposure (WCE) model adapted to case-control analyses.^43^^,^^44^ The WCE(u) exposure metric was defined as the weighted sum of predicted 25(OH)D levels at different times in the past, as follows:


(1) \begin{equation*} {\text{WCE}}_i\left({u}_i\right)=\sum_t^{u_i}w\left({u}_i-t\right){X}_i(t), \end{equation*}

where *u_i_* represents the index date for participant *i*, *X_i_(t)*, where *t ≤ u_i_ – 2* represents predicted 25(OH)D at time *t*, and the weight function *w(u_i_ − t)* assigns weights to past exposures, depending on the time *(u – t)* elapsed between their measurements and the index date, with negative weights indicating risk reduction associated with increased exposure in the corresponding time interval.^43^ Each participant contributed exposure data for the WCE model for each year from age 20 years to 2 years prior to the index date. The longest exposure history across participants was 62 years (ie, 60 years after a 2-year lag period for an 82-year-old woman), with younger participants contributing fewer years of exposure data. The numbers of invasive and borderline cases and controls who contributed any exposure data for various time intervals before the index date are provided in Table S1. The weight function was estimated using cubic regression splines, which avoided a priori assumptions regarding their shape.^43^ Separate WCE models were estimated for overall, invasive, and borderline ovarian cancer. Preliminary analyses were conducted to select the final WCE models for each outcome (Appendix S3). A 2-knot right-constrained model with a 62-year time window (ie, 60 years after a 2-year lag period) was selected for overall and invasive ovarian cancers; for borderline ovarian cancer, a 1-knot right-constrained model with a 62-year time window was used. Using the final WCE model weight function estimates, aORs (95% CIs) associated with cumulative exposure in specific time intervals in the past were computed, as described previously.^43^ We estimated 95% pointwise CIs around the weight functions and the WCE-based aORs using the Monte Carlo procedure.^43^

Of the 1406 PROVAQ study participants, we excluded 15 women (*n* = 6 cases and 9 controls) with no available exposure data and 5 women with missing information on multiple 25(OH)D predictors or confounders (*n* = 2 cases and 3 controls). Accordingly, 490 cases and 896 controls were included in the analyses. All analyses were performed using R, including customized programs for WCE analyses (R, version 4.1).^45^

## Results

The mean (SD) age of participants was 57.7 (12.1) for cases and 58.6 (12.1) for controls. Compared with controls, cases were more likely to be nulliparous, less likely to use oral contraceptives, and slightly less likely to have a university degree (Table 1). The distributions of vitamin D model predictors did not differ greatly between cases and controls (Table 1), though cases were slightly less likely to ever use vitamin D supplements. The mean predicted 25(OH)D levels over the adult lifetime were also similar between cases and controls (Table 2), but inadequate lifetime serum 25(OH)D levels (30 to < 50 nmol/L) were more frequent among cases than among controls (Table 2). Overall, women who refused to participate tended to be older, less educated, smoked more, and were more likely to have engaged in sun-seeking behaviors among both cases and controls. Among those with ovarian cancer, those who refused to participate were less likely to be nulliparous in comparison with participant cases (Table S2).

**Table 1:** Characteristics of the Prevention of Ovarian Cancer in Quebec study population, Montreal, Canada, 2011-2016.

**Characteristic**	**Controls ** **(*n* = 896)**	**All cases** **(*n* = 490)**	**Invasive cases** **(*n* = 360)**	**Borderline cases** **(*n* = 130)**
Age, no. (%), years				
<45	107 (11.9)	57 (11.6)	24 (6.7)	33 (25.4)
45 to < 55	212 (23.7)	128 (26.1)	96 (26.7)	32 (24.6)
55 to < 65	293 (32.7)	162 (33.1)	122 (33.9)	40 (30.8)
65 to < 75	197 (22.0)	103 (21.0)	84 (23.3)	19 (14.6)
≥75	87 (9.7)	40 (8.2)	34 (9.4)	6 (4.6)
Menopausal status, no. (%)^a^				
Premenopausal	294 (32.8)	166 (33.9)	109 (30.3)	57 (43.8)
Postmenopausal	602 (67.2)	324 (66.1)	251 (69.7)	73 (56.2)
Ancestry, no. (%)				
French Canadian	600 (67.0)	335 (68.4)	244 (67.8)	91 (70.0)
Other European	215 (24.0)	113 (23.0)	85 (23.6)	28 (21.5)
Other/mixed	81 (9.0)	42 (8.6)	31 (8.6)	11 (8.5)
Highest level of education completed, no. (%)				
Less than high school	82 (9.2)	53 (10.8)	42 (11.7)	11 (8.5)
High school	195 (21.8)	138 (28.2)	93 (25.8)	45 (34.6)
College/technical school	272 (30.4)	141 (28.8)	107 (29.7)	34 (26.2)
University	347 (38.7)	158 (32.2)	118 (32.8)	40 (30.8)
Parity, no. (%)				
Nulliparous	189 (21.1)	160 (32.7)	112 (31.1)	48 (36.9)
1-2	514 (57.4)	258 (52.7)	192 (53.3)	66 (50.8)
≥3	193 (21.5)	72 (14.7)	56 (15.6)	16 (12.3)
Duration of oral contraceptive use, no. (%), years^b^				
Never	169 (18.9)	105 (21.5)	89 (24.7)	16 (12.5)
<0 to < 2	155 (17.3)	93 (19.1)	65 (18.1)	28 (21.9)
2 to < 10	330 (36.8)	192 (39.3)	145 (40.3)	47 (36.7)
≥10	242 (27.0)	98 (20.1)	61 (16.9)	37 (28.9)
Adult lifetime average total MVPA, mean (SD), MET-h/wk	140.0 (95.6)	128.8 (106.4)	129.7 (103.4)	126.5 (114.9)
BMI^c^ at age 20 years, no. (%)^d^				
<25	842 (94.0)	447 (91.2)	326 (90.6)	121 (93.1)
≥25	54 (6.0)	43 (8.8)	34 (9.4)	9 (6.9)
Adult lifetime average alcohol, mean (SD), g/wk	37.8 (57.9)	40.6 (60.0)	34.6 (52.2)	57.3 (75.5)
Smoking, no. (%)^e^				
Never	418 (46.7)	196 (40.0)	155 (43.1)	41 (31.5)
Former	322 (35.9)	194 (39.6)	144 (40.0)	50 (38.5)
Current	156 (17.4)	100 (20.4)	61 (16.9)	39 (30.0)
Sun sensitivity score, mean (SD)	13.2 (2.6)	13.3 (2.6)	13.2 (2.7)	13.6 (2.5)
Adult lifetime engagement in sun-seeking behavior, no. (%)				
Yes	298 (33.3)	149 (30.4)	101 (28.1)	48 (36.9)
No	598 (66.7)	341 (69.6)	259 (71.9)	82 (63.1)
Adult lifetime vitamin D supplement use, no. (%)				
Never	258 (28.8)	159 (32.4)	111 (30.8)	48 (36.9)
Ever	638 (71.2)	331 (67.6)	249 (69.2)	82 (63.1)
Vitamin D supplement use among ever users, mean (SD), 100 IU/wk	10.0 (14.4)	8.6 (10.0)	9.0 (10.5)	7.6 (8.3)
Adult lifetime number of sun vacations, mean (SD)	7.6 (10.5)	7.4 (11.8)	6.9 (10.5)	8.7 (14.8)
Adult lifetime outdoor time with total sun protection,^f^ mean (SD), h/wk	1.2 (2.9)	1.5 (4.1)	1.3 (4.0)	1.9 (4.4)
Adult lifetime outdoor time with partial sun protection,^f^ mean (SD), h/wk	6.6 (7.7)	6.7 (9.2)	6.6 (8.6)	7.1 (10.6)
Adult lifetime outdoor time with no sun protection, mean (SD), h/wk	2.5 (4.7)	2.9 (6.1)	2.9 (6.0)	2.9 (6.1)

Abbreviations: BMI, body mass index; MVPA, moderate-to-vigorous physical activity.

^a^ Thirty-eight participants (*n* = 26 cases and 12 control participants) with missing information on menopausal status were imputed as menopausal if they reached the age of 53 years.

^b^ Two cases had missing information on oral contraceptive use.

^c^ Calculated as weight (kg) divided by square of height (m^2^).

^d^ Thirty-seven participants (*n* = 16 case and 21 control participants) had missing information on BMI at age 20 years that was imputed.

^e^ Four participants (*n* = 2 case and 2 control participants) had missing information on smoking status that was imputed.

^f^ Time spent outdoors during the summer months (May-September). Total sun protection refers to full coverage of arms and legs using clothing or sunscreen; partial protection refers to partial coverage using clothing or sunscreen.

**Table 2:** Distribution of average predicted 25(OH)D over the adult lifetime in the Prevention of Ovarian Cancer in Quebec study population, Montreal, Canada, 2011-2016.

	**Controls ** **(*n* = 896)**	**All cases** **(*n* = 490)**	**Invasive cases** **(*n* = 360)**	**Borderline cases** **(*n* = 130)**
Mean (SD), nmol/L	61.7 (9.0)	60.4 (8.9)	60.6 (8.8)	59.9 (9.0)
Range, nmol/L	36.5-104.3	37.2-86.4	37.2-86.4	38.6-84.5
Institute of Medicine classification^a^				
Adequate (50 to < 125 nmol/L), no. (%)	819 (91.4)	427 (87.1)	317 (88.1)	110 (84.6)
Inadequate (30 to <50 nmol/L), no. (%)	77 (8.6)	63 (12.9)	43 (11.9)	20 (15.4)

Abbreviation: 25(OH)D, 25-hydroxyvitamin D.

^a^ Cutpoints established by the US Institute of Medicine^32^ report in reference to serum 25(OH)D concentration and its association with bone health. No participants in our study were classified as deficient (<30 nmol/L) or concerningly high (≥125 nmol/L) in 25(OH)D concentration.

When comparing adequate versus inadequate average predicted 25(OH)D over the adult lifetime, aORs (95% CIs) were 0.65 (0.44-0.97) for overall ovarian cancer, 0.71 (0.46-1.10) for invasive cancers, and 0.52 (0.28-0.95) for borderline cancers (Table 3). The results of the multivariable fractional polynomial analyses were consistent with linear associations for continuous representations of predicted 25(OH)D (*P* > .45 for tests of linearity) and, for each 20-nmol/L increase in average predicted lifetime 25(OH)D, the odds of ovarian cancer decreased by 27% (Table 3) for overall ovarian cancer, 28% for invasive cancers, and 29% for borderline cancers. The aORs, as reported in Table 3, were not appreciably changed when BMI, which was a 25(OH)D predictor for nonsupplement users, was excluded as a covariate (results not shown). Similarly, when weighting the logistic regression analyses by the inverse probability of participation to assess potential selection bias, the aORs did not greatly change (Table S3). SIMEX corrections for measurement errors in predicted 25(OH)D suggested slightly stronger inverse associations, with estimated aORs (95% CIs) per 20-nmol/L increase in predicted 25(OH)D of 0.66 (0.62-0.70) for overall ovarian cancer, 0.64 (0.57-0.73) for invasive cancers, and 0.66 (0.61-0.71) for borderline cancers (Table S4, Figure S2). For overall ovarian cancer, there was no evidence of additive or multiplicative interactions with menopausal status, BMI, or supplement use (Table S5).

**Table 3:** Adjusted odds ratios^a^ (95% CI) for the association between average predicted 25-hydroxyvitamin D over the adult lifetime and overall ovarian cancer risk and for invasive and borderline cancers in the Prevention of Ovarian Cancer in Quebec study population, Montreal, Canada, 2011-2016.

**Predicted 25(OH)D**	**No. of controls (*n* = 896)**	**Overall ovarian cancer** **(*n* = 490)**		**Invasive ovarian cancer** **(*n* = 360)**		**Borderline ovarian cancer** **(*n* = 130)**		***P* for heterogeneity** ^**b**^
		**No.**	**aOR (95% CI)**	**No.**	**aOR (95% CI)**	**No.**	**aOR (95% CI)**	
Inadequate (<50 nmol/L)	77	63	1.00 (Referent)	43	1.00 (Referent)	20	1.00 (Referent)	
Adequate (≥50 nmol/L)	819	427	0.65 (0.44-0.97)	317	0.71 (0.46-1.10)	110	0.52 (0.28-0.95)	.24
Per 20-nmol/L increment^c^			0.73 (0.55-0.96)		0.72 (0.53-0.99)		0.71 (0.44-1.16)	.99

Abbreviations: aOR, adjusted odds ratio; 25(OH)D, 25-hydroxyvitamin D.

^a^ Adjusted for age, educational attainment, ancestry, parity, average total moderate-to-vigorous physical activity over the adult lifetime, body mass index at age 20 years.

^b^ *P* value for heterogeneity between borderline and invasive cancers assessed using a likelihood ratio test.

^c^ aORs based on continuous representation of predicted 25(OH)D. In healthy adults, 20 nmol/L corresponds to the expected increase in serum 25(OH)D with a 1000-1250 IU intake of vitamin D_3_.

Associations by histological type, stratified by invasive versus borderline tumors (Table S6), were generally similar to those observed for ovarian cancer overall, except for invasive endometrioid (*n* = 49 cases) and clear cell cancers (*n* = 23 cases), with aORs of 0.22 (0.09-0.51) and 1.80 (0.69-4.68), respectively, for each 20-nmol/L increment in predicted 25(OH)D. The aOR for the high-grade serous histotype (*n* = 241 cases) was 0.63 (0.39-1.04), comparing adequate versus inadequate predicted 25(OH)D and 0.74 (0.52-1.06) for each 20-nmol/L increase.

### WCE analysis

Both the Akaike information criterion and likelihood ratio tests comparing the WCE model with the model with unweighted cumulative exposure (corresponding to the conventional analyses of lifetime average) indicated that differential weighting of past 25(OH)D levels improved the model’s fit to the data (Akaike information criterion of 4827.1 for the WCE model versus 4830.7 for the unweighted model; likelihood ratio test *P* < .02 for overall ovarian cancer).

**Figure 1:**
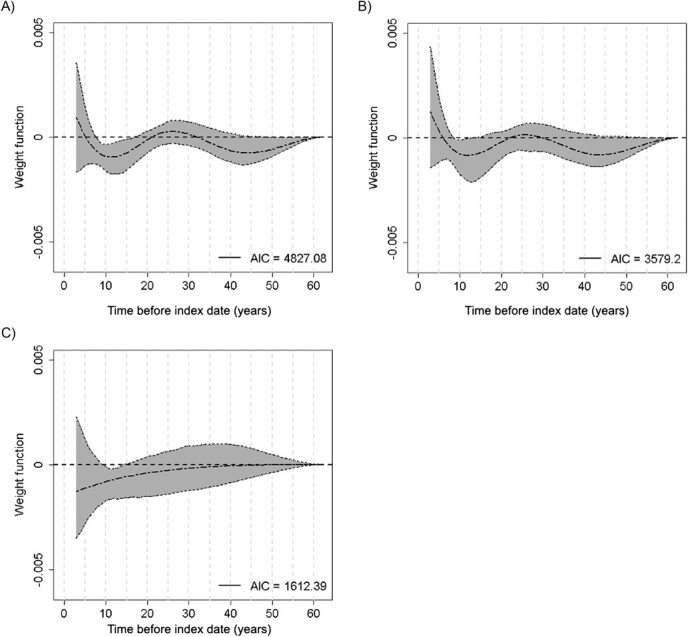
Estimated weight functions (dashed line) and pointwise 95% CIs (shaded areas) for the associations between past predicted 25(OH)D exposure and ovarian cancer for A) overall ovarian cancer, B) invasive ovarian cancer, and C) borderline ovarian cancer in the Prevention of Ovarian Cancer in Quebec (PROVAQ) study population, Montreal, Canada, 2011-2016. The weight function reflects past exposure since the index date. The index date was defined as the date of diagnosis for cases and the date of study participation for controls. Negative weight estimates indicate risk reduction (ie, an inverse association between exposure observed in the corresponding time interval in the past and risk). 25(OH)D, 25-hydroxyvitamin D; AIC, Akaike information criterion.

Consistent with the conventional analyses, the results of the WCE models for overall, invasive, and borderline cancers indicated that higher past 25(OH)D levels across the adult lifetime were associated with reduced risks, as reflected in Figure 1, by negative weights across most of the time window. However, a critical period of exposure of approximately 5 to 20 years before the index date was suggested where, for overall, invasive, and borderline cancers, the inverse associations were strongest (Figure 1). The corresponding aORs for this time window, when adjusted for the covariates and the predicted 25(OH)D in other time windows, ranged from 0.81 to 0.85 for each 20-nmol/L increment in predicted 25(OH)D level (Table 4). For overall and invasive ovarian cancers, there was an indication of another important period of 35 to 55 years prior to the index date, where the weights were low and the corresponding 95% CIs excluded or were very close to 0 (Figure 1), and the aORs were similar to those for 5 to 20 years (Table 4). Because borderline cases were younger, with only 67 cases providing exposure data for 35 years or more prior to the index date, the associations with this time window could not be reliably assessed. For the period 20 to 35 years prior to the index date, the weights were very near 0 for overall, invasive, and borderline ovarian cancers (Figure 1) and, accordingly, the corresponding aORs were close to null (Table 4).

**Table 4:** Adjusted odds ratios^a^^,^^b^ (95% CI) for the association between past time intervals of predicted 25(OH)D exposures and risk of ovarian cancer in the Prevention of Ovarian Cancer in Quebec study population, Montreal, Canada, 2011-2016.

**Timing of predicted 25(OH)D exposure, years prior to index date**	**aOR (95% CI)**		
	**Overall ovarian cancer**	**Invasive ovarian cancer**	**Borderline ovarian cancer**
5-20	0.82 (0.69-0.94)	0.85 (0.66-0.99)	0.81 (0.64-0.95)
20-35	1.03 (0.89-1.19)	0.99 (0.81-1.15)	0.94 (0.69-1.24)
35-55	0.79 (0.66-1.02)	0.78 (0.65-1.04)	

Abbreviations: aOR, adjusted odds ratio; 25(OH)D, 25-hydroxyvitamin D.

^a^ Adjusted for age, educational attainment, ancestry, parity, average total moderate-to-vigorous physical activity over the adult lifetime, body mass index at age 20 years.

^b^ aORs are for an increment of 20 nmol/L. In healthy adults, 20 nmol/L corresponds to the expected increase in serum 25(OH)D with a 1000-1250 IU intake of vitamin D_3_.

## Discussion

In this study, we found that higher average predicted 25(OH)D over the adult lifetime was associated with a reduced risk of ovarian cancer. This was observed when comparing levels considered to be adequate versus inadequate, as well as with continuous representations of predicted 25(OH)D. Furthermore, these associations were found for ovarian cancer overall and for invasive and borderline cancers, and were similar across categories of BMI, supplement use, and menopausal status. When exploring the role of predicted 25(OH)D levels in different past time intervals, a critical period of 5 to 20 years before diagnosis was suggested for overall, invasive, and borderline ovarian cancers, as well as for 35 to 55 years prior for invasive and overall cancers, for which a sufficient number of older cases allowed such a long-term assessment.

The relationship between total vitamin D exposure and ovarian cancer has been examined in 10 previous studies that directly measured serum 25(OH)D.^13^^‑^^22^ Of these, 4 suggested an inverse association.^13^^‑^^16^ However, 1 of these was a cross-sectional study of prevalent cases^15^ and possibly was affected by survival bias.^46^ Two other studies^13^^,^^14^ measured 25(OH)D levels in younger premenopausal women (median age of serum sampling, 30-31 years), suggesting that earlier periods in the life course may better capture etiologically relevant vitamin D exposure. However, cases were very young in these studies (<44 years),^13^^,^^14^ thus the results may not be reflective of the etiology of most ovarian cancers, for which the median age at diagnosis is approximately 63 years.^47^ The remaining study reporting an inverse association was conducted with an older population (mean age at baseline, 57 years) with data in the UK Biobank, with a median follow-up time of 13 years; however, the study population was restricted to those with metabolic syndrome.^16^ Among the other 6 studies, which reported null associations overall,^17^^‑^^22^ 2 observed an inverse association among overweight/obese women only,^17^^,^^18^ consistent with the findings from the UK Biobank data on women with metabolic syndrome. This is in contrast to our results, where the inverse association did not vary according to BMI either at 2 years prior to participation or averaged across the adult lifetime.

We hypothesized that the null associations reported in previous prospective studies may be partly due to relying on serum 25(OH)D measures taken during etiologically less relevant time windows.^17^^‑^^22^ In particular, blood samples were collected at ages 50 to 58 years across the studies, and the median time to ovarian cancer diagnosis was only 5 to 7 years after the measurement. However, our analyses suggest that the critical period extends back well beyond the last 5 to 7 years, with the strongest risk reductions at 10 to 15 years before the index date (Figure 1). Thus, our results suggest that the previous null findings may partly reflect exposures measured outside the etiologically relevant window.

We acknowledge that we did not directly measure serum 25(OH)D but used a predicted estimate. Only 1 previous study, of the Nurses’ Health Study (NHS) and NHS II cohorts, also used prediction models to estimate yearly 25(OH)D levels for up to 34 years of follow-up.^48^ The mean predicted 25(OH)D concentrations were similar to those in our study (~60-65 nmol/L). In the NHS II, women in the highest tertile of cumulative average predicted 25(OH)D, compared with the lowest tertile, had a lower risk of epithelial ovarian cancer (relative risk = 0.68; 95% CI, 0.48-1.00),^48^ consistent with our results. However, the association observed in the NHS cohort suggested an increased risk (relative risk, 1.27; 95% CI, 1.00-1.61).^48^ It is not clear why the associations were in opposite directions. The authors suggested differences in the distribution of age and menopausal status between the 2 cohorts and/or a cohort effect.^48^ Indeed, the participants in the PROVAQ study are in similar birth cohorts to NHS II participants. However, the inverse association we observed between predicted 25(OH)D and ovarian cancer was of similar magnitude for premenopausal and postmenopausal women. Of note is that adjustment for BMI, a predictor within the 25(OH)D prediction model for the NHS cohort, attenuated the inverse association in NHS II,^48^ in contrast to our findings.

Our results suggested another time window of reduced risk, with exposure 35 to 55 years prior to diagnosis, for overall and invasive cancers. Although we cannot rule out that vitamin D at other periods may also contribute to risk, it is possible that the exposure windows of 5 to 20 and 35 to 55 years prior may relate to etiologic windows for cancer initiation and promotion. Of note is that very recent exposure (ie, close to diagnosis) was not associated with risk. Given that no previous study, to our knowledge, has examined the potential importance of timing of past 25(OH)D exposure for ovarian cancer risk, more research is needed to confirm our findings.

The use of prediction models to assess 25(OH)D levels is a cost-efficient alternative to directly measuring vitamin D at multiple time points in epidemiologic studies. However, the use of predicted 25(OH)D implies measurement errors. Sensitivity analysis using SIMEX methodology^40^ yielded slightly stronger ORs when accounting for random measurement errors of the magnitude suggested by validation analyses of our original prediction models.^29^ Thus, the use of predicted 25(OH)D likely underestimated associations between 25(OH)D and ovarian cancer risk, as we expected. In addition, although the interview questions to assess recent vitamin D predictors in our study were validated against serum 25(OH)D levels,^29^ we cannot rule out errors in recalling values of some relevant predictors over many decades. Indeed, participants were asked to recall outdoor activities and lifestyle behaviors (eg, sun vacations, alcohol intake, physical activity) and changes in these variables across adulthood. To assist with this recall, we used a life-events calendar,^31^ and our questionnaire is similar to validated questionnaires designed to collect information on lifetime lifestyle behaviors in epidemiologic studies.^49^^,^^50^ Although some recall errors are unavoidable, given the wide array of information asked, we expect the resulting errors in predicted 25(OH)D levels to be nondifferential, resulting in some bias toward the null.

With response rates of 56% among controls and 78% among cases, the participants might not fully represent the source population. In a sensitivity analysis, we used inverse probability of participation weighting to account for potential selection bias. Although women who refused to participate tended to be older, had lower education levels, smoked more, and reported more sun-seeking behaviors, the main results did not change substantially. However, other unknown, and thus unmeasured, factors may be associated with nonparticipation, limiting this sensitivity analysis. Thus, we cannot rule out that residual selection bias might have affected some of our findings. Finally, although we considered a number of potential confounders in our directed acyclic graphs, we cannot rule out the possibility of uncontrolled confounding due to unknown and unmeasured variables, given the limited knowledge on ovarian cancer etiology.

In summary, higher predicted 25(OH)D over the adult lifetime was associated with a reduced risk of ovarian cancer. Our findings highlight the importance of measuring 25(OH)D exposure throughout the entire adulthood period and suggest the impact may be more important in certain periods. Future studies should consider how timing of 25(OH)D exposure relates to ovarian cancer risk.

## Acknowledgments

Results from this article were presented as oral presentations at the Society for Epidemiologic Research conference, Portland, Oregon, June 2023; and the Canadian Society for Epidemiology and Biostatistics conference, Halifax, NS, Canada, June 2023.

## Supplementary Material

Web_Material_kwae070

## Data Availability

The data that support the findings of our study are available from the corresponding author upon reasonable request and institutional approval. Computing code that supports the findings of this study is publicly available at https://osf.io/y586p/.
