# Supplementary material for: Predicted 25-hydroxyvitamin D over the adult lifetime and the risk of ovarian cancer
Source: Am J Epidemiol. 2024 May 17;193(9):1233–41. doi: 10.1093/aje/kwae070 (PMC11369219; doi:10.1093/aje/kwae070)
Supplement: Web_Material_kwae070 [file web_material_kwae070.docx]

**Supplementary Data**

**Title:** Predicted 25-hydroxyvitamin D over the adult life and the risk of ovarian cancer

**Authors:** Jennifer A. Ritonja, Coraline Danieli, Magnoudewa Priscille Pana, Michael J. Palmer, Kevin L’Espérance, Vikki Ho, Michal Abrahamowicz, Anita Koushik

Table of Contents

[Appendix S1: Description of 25(OH)D Prediction Models 2](#_Toc148531004)

[Application of 25(OH)D prediction models, for vitamin D supplement users 2](#_Toc148531005)

[Application of 25(OH)D prediction models, for vitamin D supplement non-users 3](#_Toc148531006)

[Figure S1: Directed acyclic graph for the association between adult lifetime 25(OH)D and ovarian cancer risk. 4](#_Toc148531007)

[Appendix S2: Sensitivity analysis on potential selection bias 5](#_Toc148531008)

[Table S1: Numbers of invasive and borderline cases and controls who provided any exposure data for various time intervals prior to the index date. 6](#_Toc148531009)

[Appendix S3: Preliminary weighted cumulative exposure (WCE) analyses to select the final WCE models for each of the outcomes 7](#_Toc148531010)

[Comparison of alternative WCE models based on the number of knots^1^ 7](#_Toc148531011)

[Comparison of alternative WCE models based on the maximum time window of exposure 8](#_Toc148531012)

[Comparison of alternative WCE models based on the constraint of the weight function 8](#_Toc148531013)

[Table S2. Comparison of women who refused to participate with those that participated 9](#_Toc148531014)

[Table S3. Comparison of aORs^1^ (95% CI) from the main analysis and the analysis weighted by the inverse-probability of participation (IPPW) 10](#_Toc148531015)

[Table S4. Comparison of aORs^1^ (95% CI) from the main analysis and after accounting for measurement error using simulation-extrapolation (SIMEX) 11](#_Toc148531016)

[Figure S2. Graphical illustration of the simulation-extrapolation (SIMEX) correction process for 25(OH)D measurement errors 12](#_Toc148531017)

[Table S5. Interaction between predicted 25(OH)D and menopausal status, body mass index (BMI) and vitamin D supplement use 13](#_Toc148531018)

[Table S6. Adjusted ORs^1^ (95% CI) for the association between average predicted 25(OH)D over the adult lifetime and ovarian cancer risk by histological type and by borderline/invasive status 14](#_Toc148531019)

# **Appendix S1: Description of 25(OH)D Prediction Models**

In a previous study, we developed and validated multivariable least squares regression models to predict circulating 25-hydroxyvitamin D [25(OH)D].^1^ Prediction models were built for users and non-users of vitamin D supplements separately. More details on how the prediction models were built, including cross-validation of the models, can be found in the original publication by Ho et al (2018).^1^ Pertinent details of the prediction models, including the model weights (i.e. regression coefficients), how predictors were operationalized in the models, and how missing data were handled for each predictor are presented in the tables below, first for supplement users and next for non-users.

##

## **Application of 25(OH)D prediction models, for vitamin D supplement users**

| **Predictor** | **Weight (model coefficient)** | **Operationalization of predictor** | **Description** |
| --- | --- | --- | --- |
| Intercept | 51.87 |  |  |
| Alcohol intake | 0.08 | Continuous, in grams per week | Participants reported the quantity, dose and frequency of alcohol intake across adulthood from four sources: beer, red wine, white wine, and spirits.^2^ Total quantity in grams per week was calculated. Median weekly alcohol intake among controls was imputed for 3 participants (1 case, 2 controls) with missing information. |
| Outdoor sun exposure with partial sun protection | 0.12 | Continuous, in hours per week | Defined as the number of hours per week spent outdoors with arms and legs partially covered by clothing or sunscreen, during work, commuting, and leisure activities across adulthood. Only time spent outdoors during the summer months (May to September) was considered. All participants had complete information. |
| Vitamin D supplement dose | 0.13 | Continuous, in 100 International Units per week | Participants reported the quantity, dose and frequency of vitamin D supplement use across adulthood from: vitamin D, on its own or as part of calcium supplements, multivitamins, cod liver oil in capsules or tablets, and cod liver oil in liquid. Total weekly intake was calculated and then scaled to 100 International Units per week. 39 participants (15 cases, 24 controls) did not recall whether they ever took vitamin D, multivitamins, and/or cod liver oil supplements; we imputed the modal value among controls of the corresponding supplement type, which was never use for all supplement types. 30 participants (9 cases,18 controls) who reported taking cod liver oil in capsules or tablets and 65 participants (30 cases, 35 controls) who reported taking vitamin D on its own or as part of calcium supplements had missing dose information, which was imputed by the respective median value among users of the corresponding supplement type. |
| Menopausal status | 24.62 | Categorized:  0=Premenopausal vs.  1=Postmenopausal | Defined according to whether or not participants had reported menstruating two years prior to study participation. 38 participants (12 cases, 26 controls) were missing information on menopausal status and age at menopause. These participants were assumed to be menopausal if they had reached the age of 53 (and thus, age at menopause was imputed as 53). 24 postmenopausal women (13 cases, 11 controls) were missing information on age at menopause, and thus age at menopause was imputed as 53. |
| Vacation with sun protection | 21.56 | Categorized:  0=No vacation vs.  “Vacation rate” (during years of vacation(with sun protection) | Defined as a vacation during the winter to a summer climate with sun protection (arms/legs covered by clothing or sunscreen, or they stayed in shade). As originally reported,^1^ this predictor is part of a 3-level categorical variable (with the predictor below): no vacation (reference), vacation with sun protection, and vacation with partial or no sun protection. In the full data set, we only had information on lifetime number of sun vacations and the ages of first and last taken vacations, rather than exact times when each vacation occurred. Thus, a “vacation rate” was calculated as the total number of vacations divided by the number of years from first vacation to last vacation. which was attributed for each year during periods when sun vacations were taken (with sun protection). For this predictor level and the following, a value of 0 was given for periods when sun vacations were not taken. All participants had complete information. |
| Vacation with partial or no sun protection | -14.97 | As above, but for vacation years with partial/no protection | Defined as a vacation during the winter to a summer climate with partial (arms and legs partially covered by clothing or by sunscreen) or no sun protection. “Vacation rate” as defined above. All participants had complete information. |

## **Application of 25(OH)D prediction models, for vitamin D supplement non-users**

| **Predictor** | **Weight (model coefficient)** | **Operationalization of predictor** | **Description** |
| --- | --- | --- | --- |
| Intercept | 102.72 |  |  |
| Body mass index (BMI) | -0.95 | Continuous, in kg/m^2^ | Participants reported their tallest attained height, body weight and body silhouette^3^ at ages 20, 30, 40, 50, 60 and 70, where applicable, and at to two years prior to study participation. Body weight and height were used to calculate BMI for each year of adult life (in kg/m^2^). To estimate BMI for ages between the decade years where weight was reported (e.g. ages 21, 22, etc.), we created a linear slope between reported BMI measures to interpolate measures that accounted for incremental increases/decreases in BMI over time. If weight or height was not reported, we imputed BMI using the median BMI value among controls who reported similar body silhouettes. 3 participants (1 case, 2 controls) had missing information on height, 14 participants (6 cases, 8 controls) had missing information on weight for one age period, and 32 participants (12 cases, 20 controls) had missing information on weight for 2 or more age periods. |
| Sun sensitivity score | -2.22 | Continuous | Calculated as the sum of responses to self-described eye colour, hair colour, tendency to burn at first exposure to the sun, tanning ability, and skin tone, with range in score from 5 (highest sun sensitivity) to 19 (lowest sun sensitivity).^4^ Hot deck imputation was used to impute information on tanning ability, skin tone and tendency to burn for 45 participants with missing information. |
| Season of blood collection | 8.67 | 0.5 for all years | In the original paper^1^, this variable referred to the season serum blood was collected for a participant, and was categorized as 0 = October to March, 1 = April to September. Since this variable is not relevant for average yearly exposure, a value of 0.5 was assigned for all years. |
| Vacation with sun protection | 18.75 | Categorized:  0=No vacation vs.  “Vacation rate” (during years of vacation with sun protection) | Defined as a vacation during the winter to a summer climate with sun protection (arms and legs covered by clothing, they wore sunscreen, or they stayed in shade). “Vacation rate” as defined above. All participants had complete information. |
| Vacation with partial or no sun protection | 14.68 | As above, but for vacation years with partial/no protection | Defined as a vacation during the winter to a summer climate with partial (arms and legs partially covered by clothing or by sunscreen) or no sun protection. “Vacation rate” as defined above. All participants had complete information. |

**References**

1. Ho V, Danieli C, Abrahamowicz M, et al. Predicting serum vitamin D concentrations based on self-reported lifestyle factors and personal attributes. *Br J Nutr*. 2018;120(7):803-812.

2. L'Esperance K, Grundy A, Abrahamowicz M, et al. Alcohol intake and the risk of epithelial ovarian cancer. *Cancer Causes Control.* 2023;34(6):533-541.

3. Stunkard AJ, Sørensen T, Schulsinger F. Use of the Danish Adoption Register for the study of obesity and thinness. *Res Publ Assoc Res Nerv Ment Dis*. 1983;60:115-20.

4. Tacke J, Dietrich J, Steinebrunner B, Reifferscheid A. Assessment of a new questionnaire for self-reported sun sensitivity in an occupational skin cancer screening program. *BMC Dermatol*. 2008;8:4.

# **Figure S1: Directed acyclic graph for the association between adult lifetime 25(OH)D and ovarian cancer risk.**

We selected the most parsimonious minimally sufficient set of adjustment variables which included age (frequency-matching variable), highest level of education attained, ancestry, parity, average total moderate-to-vigorous physical activity over the adult lifetime, and body mass index. Given that experimental studies indicate that 25(OH)D may also have a role in adipogenesis and adipocyte metabolism,^1,2^ body mass index at the earliest time point available (i.e., at age 20) was used to represent body mass index as a confounder, in order to reduce the potential for bias by conditioning on a possible mediator (i.e., BMI at a later period).^3^

**
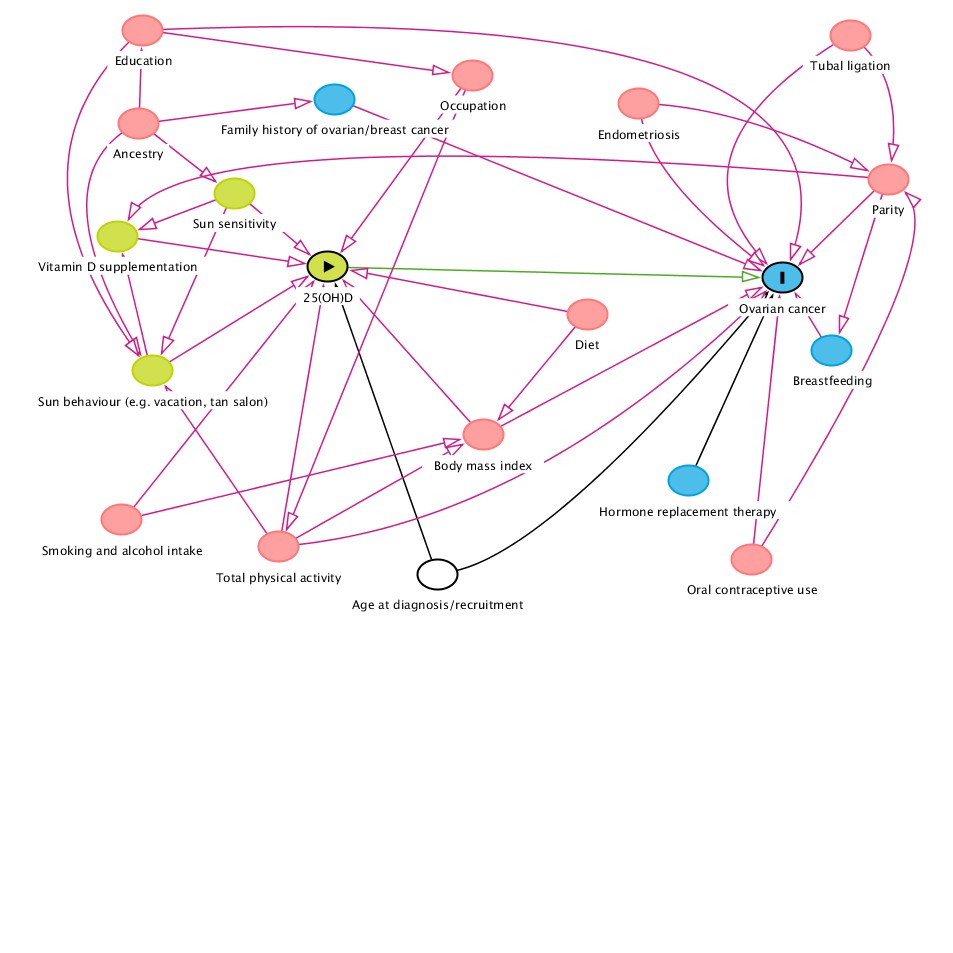

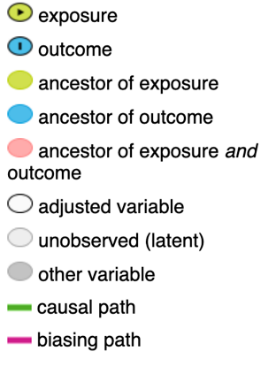
**

##

**References**

1. Ruiz-Ojeda FJ, Anguita-Ruiz A, Leis R, Aguilera CM. Genetic Factors and Molecular Mechanisms of Vitamin D and Obesity Relationship. *Ann Nutr Metab*. 2018;73(2):89-99. doi: 10.1159/000490669.

2. Migliaccio S, Di Nisio A, Magno S, Romano F, Barrea L, Colao AM, Muscogiuri G, Savastano S. Vitamin D deficiency: a potential risk factor for cancer in obesity? *Int J Obes (Lond).* 2022;46(4):707-717. doi: 10.1038/s41366-021-01045-4.

3. Lash T, VanderWeele T, Haneuse S, Rothman K. *Modern Epidemiology, 4th edition*. Lippincott Williams & Wilkins; 2021.

# **Appendix S2: Sensitivity analysis on potential selection bias**

Given the non-participation rates of 22% for cases and 44% for controls, we assessed the possible influence of selection bias by weighting our main analyses by the inverse-probability of study participation. Using a brief questionnaire, we obtained information on age (continuous), education attainment (<high school, high school, college/technical, university), parity (nulliparous, 1, 2, ≥3 births), smoking status (never, former, current smoker), years of smoking (continuous) and engagement in sun-seeking behavior (yes, no) from women who refused to participate. Engagement in sun-seeking behavior was determined using the question of “Do you in general like to seek out the sun?”. These questions were also in the main interview and thus available for participants.

In total, 449 (64%) women who refused to participate completed the brief questionnaire (62 cases (59%), 387 controls (68%)). Missing information was minimal; among those who participated, 3 cases and 3 controls had missing information on smoking history. Among those who refused to participate, 7 controls and 2 cases had missing information on smoking history, pregnancy or sun seeking behavior. Modal values among controls were imputed for categorical variables and median values among controls were imputed for continuous variables.

A logistic regression model, with participation (yes/no) as the binary outcome and the aforementioned variables as predictors, was fit for cases and controls separately. The results were then used to calculate the predicted probability of participation for each participant. We then weighted the main logistic regression analyses, with a robust variance estimator, by the inverse of the individual predicted probabilities of participation.^1,2^

**References**

1. Hernán MA, Hernandez-Diaz S, Robins JM. A structural approach to selection bias. *Epidemiol.* 2004;15(5):615-25.

2. Hernán MA, Robins JM. *Causal Inference: What If*. Chapman & Hall/CRC; 2020.

**Table S1: Numbers of invasive and borderline cases and controls who provided any exposure data for various time intervals prior to the index date.**

| **Time Interval** | **Number of Controls** | **Number of Invasive Cases** | **Number of Borderline Cases** |
| --- | --- | --- | --- |
| 2-10 years prior to index date^1^ | 896 | 360 | 130 |
| 11-20 years prior to index date | 875 | 356 | 121 |
| 21-30 years prior to index date | 837 | 349 | 106 |
| 31-40 years prior to index date | 726 | 294 | 85 |
| 41-50 years prior to index date | 445 | 185 | 43 |
| 51-62 years prior to index date | 181 | 76 | 14 |

^1^ Exposure data two years prior to the index date were not included in our analyses to account for latency between disease initiation and diagnosis.

**Appendix S3: Preliminary weighted cumulative exposure (WCE) analyses to select the final WCE models for each of the outcomes (i.e., ovarian cancer overall and by tumor behavior)**

***General Approach***

In preliminary weighted cumulative exposure (WCE) analyses to select the final WCE model, we addressed: (1) the number of knots; (2) the maximum time window over which past 25(OH)D exposure may be associated with ovarian cancer risk; and, (3) the constraint of weight function, in that order. Cancer is a chronic disease for which induction periods for many exposures can be expected to be long, with very recent exposures unlikely to play a causal role. It is for this reason that the last two years were disregarded in the calculation of average predicted 25(OH)D over the adult lifetime, as described in the main text. This two-year lag period was thus also incorporated into all WCE analyses.

The longest possible time window of exposure in the study population was 62 years. However, not all participants had data up to 62 years, given the varying ages at diagnosis/study participation. Given that prior knowledge about the mechanisms of 25(OH)D on cancer risk is not sufficient to formally determine how long ago past exposure may affect a current health outcome, we considered both a right-constrained WCE model, where the weight function was forced to decay to zero at the right end, implying no association at the distant past, and an unconstrained model which imposes no hypotheses to the ends of the interval (i.e., very recent or distant past).

Thus, the choice of the number of knots was addressed for 25(OH)D exposures accumulated over the longest possible time window in our study population of 62 years (60 years after a 2-year lag period), where potential benefits of added flexibility in modeling the weight function should be most evident. Next, to address the uncertainty regarding how long ago past 25(OH)D exposures may be associated with the current risk of ovarian cancer, we fitted alternative right-constrained and unconstrained WCE models, using the number of knots determined in the first step, for shorter time windows of 22 and 42 years. Finally, we addressed the constraint of the weight function. In this step, we also considered a left constrained model, even though the two-year lag was already incorporated.

***Step 1: Number of knots***

For ovarian cancer overall and the invasive type, the 2-knot right-constrained model yielded a better fit than the simpler 1-knot and the more complex 3-knot models, as seen in the AIC comparisons and likelihood ratio tests in the Table below. This was observed for the right-constrained model as well as for the unconstrained model, which imposes no hypothesis to the ends of the support interval (very recent or very distant exposures). For borderline ovarian cancer, the 1-knot models yielded a better fit than the more complex 2-knot or 3-knot models.

## **Comparison of alternative WCE models based on the number of knots^1^**

|  | **Right constrained, 62-year time window** | | | **Unconstrained, 62-year time window** | | |
| --- | --- | --- | --- | --- | --- | --- |
|  | **Overall** | **Invasive** | **Borderline** | **Overall** | **Invasive** | **Borderline** |
| **AIC for model with 1 knot** | 4832.513 | 3582.006 | 1612.39 | 4829.296 | 3581.415 | 1615.013 |
| **AIC for model with 2 knots** | 4827.084 | 3579.204 | 1614.381 | 4827.363 | 3579.657 | 1616.298 |
| **AIC for model with 3 knots** | 4827.877 | 3579.645 | 1616.238 | 4828.577 | 3581.518 | 1618.379 |
|  |  |  |  |  |  |  |
| ***p,* LRT for 2 knots vs 1 knot** | 0.006 | 0.028 | 0.924 | 0.047 | 0.053 | 0.398 |
| ***p*, LRT for 3 knots vs 1 knot** | 0.013 | 0.042 | 0.927 | 0.095 | 0.142 | 0.728 |
| ***p*, LRT for 2 knots vs 3 knots** | 0.272 | 0.212 | 0.705 | 0.375 | 0.709 | 1.000 |

^1^ The AIC for the null model that excludes the predicted 25(OH)D exposure variable was 4845.907 in the overall model, 1640.403 in the borderline model, and 3626.429 in the invasive model.

***Step 2: Maximum time window of 25(OH)D exposure***

In the second step, we fitted alternative 2-knot right-constrained and unconstrained WCE models for time windows of 22 and 42 years for both overall and invasive ovarian cancer (see table below). For borderline ovarian cancer, we fitted alternative 1-knot right-constrained and unconstrained WCE models for the same time windows. For all three of overall, invasive and borderline ovarian cancers, the 62-year models yielded the lowest AIC values.

## **Comparison of alternative WCE models based on the maximum time window of exposure**

|  | **Right constrained** | | | **Unconstrained** | | |
| --- | --- | --- | --- | --- | --- | --- |
|  | **Overall^1^** | **Invasive^1^** | **Borderline^2^** | **Overall^1^** | **Invasive^1^** | **Borderline^2^** |
| **AIC, 62-year time window** | 4827.084 | 3579.204 | 1612.390 | 4827.363 | 3579.657 | 1615.013 |
| **AIC, 42-year time window** | 4830.334 | 3581.532 | 1612.370 | 4829.776 | 3579.615 | 1616.037 |
| **AIC, 22-year time window** | 4830.839 | 3580.879 | 1612.267 | 4833.721 | 3582.775 | 1614.248 |

^1^ Model with 2 knots, as identified in step 1.

^2^ Model with 1 knot, as identified in step 1.

***Step 3: Constraint of weight function***

In the third step considering the constraint of the weight function, the results showed that for overall, invasive and borderline cancers, unconstrained and left-constrained models did not improve the fit in comparison with the right-constrained models (see table below).

## **Comparison of alternative WCE models based on the constraint of the weight function**

|  | **Overall^1^** | **Invasive^1^** | **Borderline^2^** |
| --- | --- | --- | --- |
|  |  |  |  |
| **AIC, right constrained model** | 4827.084 | 3579.204 | 1612.390 |
| **AIC, unconstrained model** | 4827.363 | 3579.657 | 1615.013 |
| **AIC, left constrained model** | 4833.420 | 3581.936 | 1615.257 |

^1^ Model with 2 knots and 62-year time window, as identified in steps 1 and 2.

^2^ Model with 1 knot and 62-year time window, as identified in steps 1 and 2.

***Summary***

Following these preliminary analyses, all further analyses were limited to 2-knot right-constrained models with a 62-year time-window for overall and invasive ovarian cancers, while for borderline ovarian cancer, a 1-knot right-constrained model with a 62-year time-window was used.

**Table S2. Comparison of women who refused to participate with those that participated in the PROVAQ study population, Montreal, Canada (2011-2016).**

|  | **Cases** | | **Controls** | |
| --- | --- | --- | --- | --- |
|  | **Refusers^1^**  **(N=62)** | **Participants^2^**  **(N=490)** | **Refusers^1^**  **(N=387)** | **Participants^2^**  **(N=896)** |
| Age, mean (SD) | 62.5 (11.3) | 57.7 (12.1) | 61.7 (12.1) | 58.6 (12.1) |
| Highest level of education completed, n (%) |  |  |  |  |
| <High school | 17 (27.4) | 53 (10.8) | 75 (19.4) | 82 (9.2) |
| High school | 23 (37.1) | 138 (28.2) | 147 (38.0) | 195 (21.8) |
| College/technical | 8 (12.9) | 141 (28.8) | 75 (19.4) | 272 (30.4) |
| University | 14 (22.6) | 158 (32.2) | 90 (23.3) | 347 (38.7) |
| Parity, n (%) |  |  |  |  |
| Nulliparous | 14 (22.6) | 160 (32.7) | 80 (20.7) | 189 (21.1) |
| 1-2 | 38 (61.3) | 258 (52.7) | 206 (53.2) | 514 (57.4) |
| ≥3 | 10 (16.1) | 72 (14.7) | 101 (26.1) | 193 (21.5) |
| Smoking status, n (%) |  |  |  |  |
| Never | 18 (29.0) | 196 (40.0) | 177 (45.7) | 418 (46.7) |
| Former | 21 (33.9) | 194 (39.6) | 137 (35.4) | 322 (35.9) |
| Current | 23 (37.1) | 100 (20.4) | 73 (18.9) | 156 (17.4) |
| Smoking duration in years (mean, SD) | 20.7 (19.5) | 15.0 (16.1) | 13.9 (17.5) | 13.9 (17.0) |
| Engagement in sun-seeking behavior^3^ |  |  |  |  |
| Yes | 32 (51.6) | 149 (30.4) | 172 (44.4) | 298 (33.3) |
| No | 30 (48.4) | 341 (69.6) | 215 (55.6) | 598 (66.7) |

^1^ 7 controls and 2 cases who refused to participate had missing information on smoking history, pregnancy or sun seeking behavior. Modal values among controls were imputed for categorical variables and median values among controls were imputed for continuous variables.

^2^ 3 cases and 3 controls who participated had missing information on smoking history. Modal values among controls were imputed for categorical variables and median values among controls were imputed for continuous variables.

^3^ Based on the question, “Do you in general like to seek out the sun?”.

**Table S3. Comparison of aORs^1^ (95% CI) from the main analysis and the analysis weighted by the inverse-probability of participation (IPPW), for 25(OH)D and overall, invasive and borderline ovarian cancers in the PROVAQ study population, Montreal, Canada (2011-2016).**

| **Ovarian cancer type** | **Predicted 25(OH)D** | **aOR (95% CI)^2^** | |
| --- | --- | --- | --- |
|  |  | **Main analysis^3^** | **IPPW analysis** |
| Overall | Adequate vs. Inadequate | 0.65 (0.44-0.97) | 0.66 (0.44-0.98) |
|  | Per 20 nmol/L | 0.73 (0.55-0.96) | 0.72 (0.55-0.95) |
|  |  |  |  |
| Invasive | Adequate vs. Inadequate | 0.71 (0.46-1.10) | 0.71 (0.45-1.10) |
|  | Per 20 nmol/L | 0.72 (0.53-0.99) | 0.72 (0.53-0.98) |
|  |  |  |  |
| Borderline | Adequate vs. Inadequate | 0.52 (0.28-0.95) | 0.55 (0.30-0.99) |
|  | Per 20 nmol/L | 0.71 (0.44-1.16) | 0.71 (0.44-1.16) |

25(OH)D, 25-hydroxyvitamin D; 95% CI, 95% confidence interval; aOR, adjusted odds ratio; IPPW, inverse-probability of participation.

^1^ Adjusted for age, educational attainment, ancestry, parity, average total moderate-to-vigorous physical activity over the adult lifetime, BMI at age 20.

^2^ aORs are for an increment of 20 nmol/L. In healthy adult individuals, a 20 nmol/L increase in serum 25(OH)D is roughly associated with a 1000-1250 IU intake of vitamin D^3^.

^3^ aORs from the main analysis as presented in Table 3.

**Table S4. Comparison of aORs^1^ (95% CI) from the main analysis and after accounting for measurement error using simulation-extrapolation (SIMEX), for 25(OH)D and overall, invasive and borderline ovarian cancers in the PROVAQ study population, Montreal, Canada (2011-2016).**

| **Ovarian cancer type** | **aOR (95% CI)^2^** | |
| --- | --- | --- |
|  | **Main analysis^3^** | **SIMEX results^4^** |
| Overall | 0.73 (0.55-0.96) | 0.66 (0.62-0.70) |
| Invasive | 0.72 (0.53-0.99) | 0.64 (0.57-0.73) |
| Borderline | 0.71 (0.44-1.16) | 0.66 (0.61-0.71) |

25(OH)D, 25-hydroxyvitamin D; 95% CI, 95% confidence interval; aOR, adjusted odds ratio, SIMEX, simulation-extrapolation.

^1^ Adjusted for age, educational attainment, ancestry, parity, average total moderate-to-vigorous physical activity over the adult lifetime, BMI at age 20.

^2^ aORs are for an increment of 20 nmol/L. In healthy adult individuals, a 20 nmol/L increase in serum 25(OH)D is roughly associated with a 1000-1250 IU intake of vitamin D_3_.

^3^ aORs from the main analysis as presented in Table 3.

^4^ 95% CIs computed from bootstrap standard errors based on 100 replicates of the SIMEX-corrected aOR.

**Figure S2. Graphical illustration of the simulation-extrapolation (SIMEX) correction process for 25(OH)D measurement errors for A) overall ovarian cancer, B) invasive ovarian cancer, and C) borderline ovarian cancer in the PROVAQ study population, Montreal, Canada (2011-2016)**. In the SIMEX procedure, increasing increments of measurement error in predicted 25(OH)D were simulated, and new resulting ORs were calculated. We then fit a linear model between the added variance and the estimated aORs, and then extrapolated this relation back to where there is no measurement error (variance=0), the intercept of which gives the aOR corrected for potential measurement error. 25(OH)D, 25-hydroxyvitamin D; aOR, adjusted odds ratio; SD, standard deviation; SIMEX, simulation-extrapolation.

**
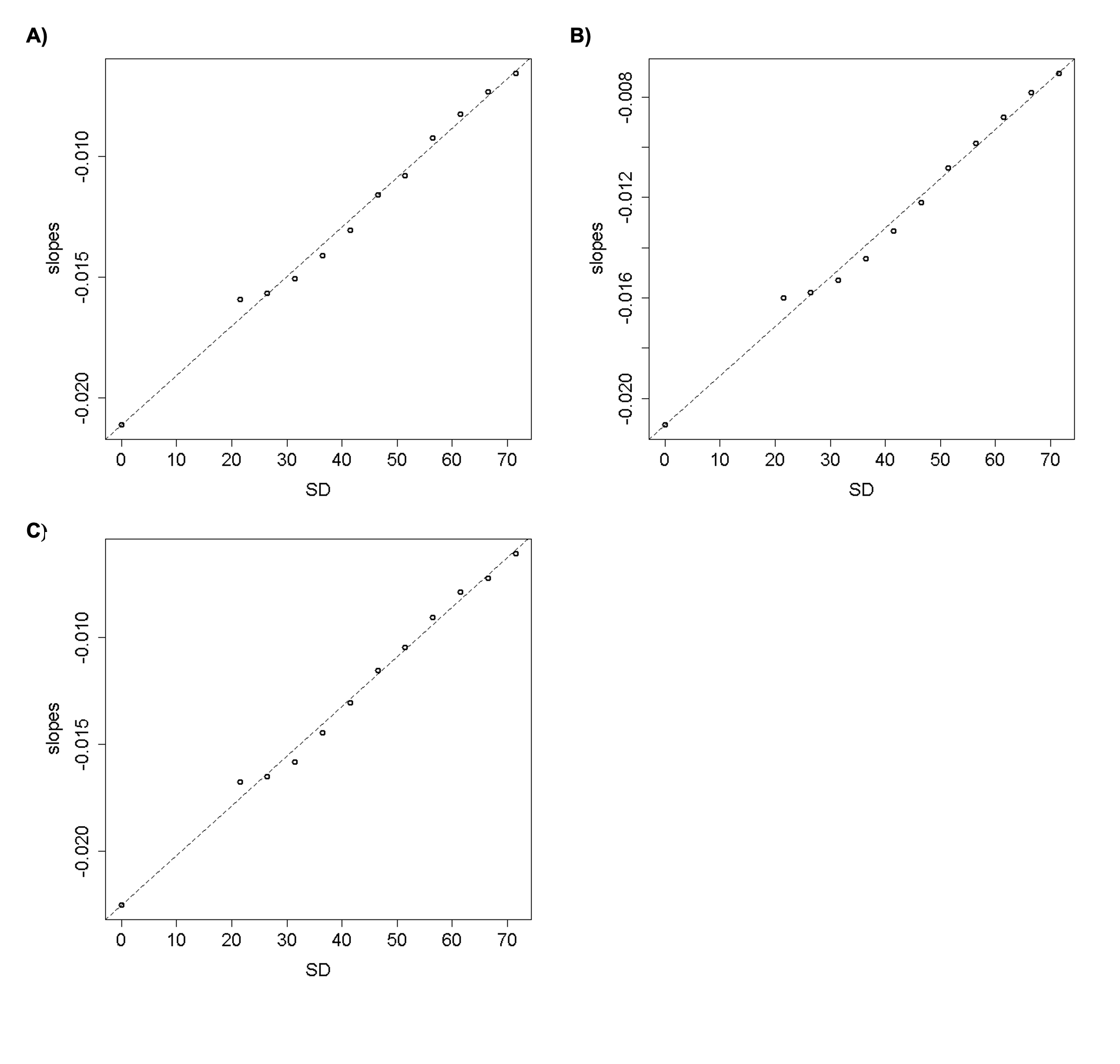
**

**Table S5. Interaction between predicted 25(OH)D and menopausal status, body mass index (BMI) and vitamin D supplement use on risk of overall ovarian cancer in the PROVAQ study population, Montreal, Canada (2011-2016).**

|  | **N_cases_/**  **N_controls_** | **aOR^1^ (95% CI)** | **N_cases_/**  **N_controls_** | **aOR^1^ (95% CI)** |
| --- | --- | --- | --- | --- |
| **Menopausal status^3^** | | | | |
|  | **Premenopausal** | | **Postmenopausal** | |
| Inadequate (<50 nmol/L) | 30/36 | 1.0 (ref) | 33/41 | 0.99 (0.48-2.08) |
| Adequate (≥50 nmol/L) | 136/258 | 0.64 (0.36-1.12) | 291/561 | 0.66 (0.36-1.21) |
| aOR^1^ (95% CI) for adequate vs. inadequate 25(OH)D within strata of menopausal status |  | 0.64 (0.36-1.12) |  | 0.66 (0.40-1.10) |
| Multiplicative interaction parameter^2^ | 1.04 (0.50-2.16) | | | |
| RERI^3^ | 0.02 (-0.68-0.73) | | | |
| **Average BMI across the adult lifetime** | | | | |
|  | **BMI<25 kg/m^2^** | | **BMI**≥**25 kg/m^2^** | |
| Inadequate (<50 nmol/L) | 16/22 | 1.0 (ref) | 47/55 | 1.04 (0.47-2.30) |
| Adequate (≥50 nmol/L) | 320/616 | 0.63 (0.39-1.02) | 107/203 | 0.67 (0.42-1.07) |
| aOR^1^ (95% CI) for adequate vs. inadequate 25(OH)D within strata of BMI |  | 0.63 (0.39-1.02) |  | 0.65 (0.32-1.32) |
| Multiplicative interaction parameter^2^ | 1.04 (0.45-2.37) | | | |
| RERI^3^ | 0.01 (-0.81-0.83) | | | |
| **BMI two years prior to recruitment/diagnosis** | | | | |
|  | **BMI<25 kg/m^2^** | | **BMI**≥**25 kg/m^2^** | |
| Inadequate (<50 nmol/L) | 12/14 | 1.0 (ref) | 51/63 | 0.94 (0.38-2.29) |
| Adequate (≥50 nmol/L) | 222/438 | 0.61 (0.26-1.40) | 205/381 | 0.63 (0.27-1.45) |
| aOR^1^ (95% CI) for adequate vs. inadequate 25(OH)D within strata of BMI |  | 0.61 (0.26-1.40) |  | 0.67 (0.43-1.04) |
| Multiplicative interaction parameter^2^ | 1.10 (0.44-2.76) | | | |
| RERI^3^ | 0.08 (-0.75-1.56) | | | |
| **Ever vs. never vitamin D supplement users^3^** | | | | |
|  | **Never user** | | **Ever user** | |
| Inadequate (<50 nmol/L) | 42/48 | 1.0 (ref) | 21/29 | 0.78 (0.38-1.60) |
| Adequate (≥50 nmol/L) | 117/210 | 0.59 (0.36-0.98) | 310/609 | 0.60 (0.38-0.96) |
| aOR^1^ (95% CI) for adequate vs. inadequate 25(OH)D within strata of supplement use |  | 0.59 (0.36-0.98) |  | 0.77 (0.41-1.44) |
| Multiplicative interaction parameter^2^ | 1.30 (0.60-2.80) | | | |
| RERI^3^ | 0.23 (-0.36-0.81) | | | |

25(OH)D, 25-hydroxyvitamin D; 95% CI, 95% confidence interval; aOR, adjusted odds ratio; BMI, body mass index; RERI, relative excess risk due to interaction.

^1^ Adjusted for age, educational attainment, ancestry, parity, average total moderate-to-vigorous physical activity over the adult lifetime, BMI at age 20.

^2^ Measure of interaction on multiplicative scale.

^3^ Measure of interaction on additive scale.

**Table S6. Adjusted ORs^1^ (95% CI) for the association between average predicted 25(OH)D over the adult lifetime and ovarian cancer risk by histological type and by borderline/invasive status^2^ in the PROVAQ study population, Montreal, Canada (2011-2016).**

| **Histological type** | **Invasive ovarian cancer** | | **Borderline ovarian cancer** | |
| --- | --- | --- | --- | --- |
|  | **Number of cases** | **aOR (95% CI)^3^** | **Number of cases** | **aOR (95% CI)^3^** |
| Serous | 257 | 0.74 (0.52-1.05) | 70 | 0.83 (0.45-1.54) |
| Mucinous | 14 | 0.72 (0.17-3.11) | 45 | 0.65 (0.29-1.44) |
| Endometrioid | 49 | 0.22 (0.09-0.51) | 3 | ----^4^ |
| Clear cell | 23 | 1.80 (0.69-4.68) | 0 | ----^4^ |

25(OH)D, 25-hydroxyvitamin D; 95% CI, 95% confidence interval; aOR, adjusted odds ratio.

^1^ Adjusted for age, educational attainment, ancestry, parity, average total moderate-to-vigorous physical activity over the adult lifetime, BMI at age 20.

^2^ Multivariable polytomous logistic regression was used to estimate aORs for histological types, stratifying by invasive and borderline ovarian cancer.

^3^ aORs are for an increment of 20 nmol/L. In healthy adult individuals, a 20 nmol/L increase in serum 25(OH)D is roughly associated with a 1000-1250 IU intake of vitamin D_3_.

^4^ Not estimated given the number of cases.
